# Meaning in military service among reservists: Measuring the effect of prosocial motivation in a moderated-mediation model

**DOI:** 10.3389/fpsyg.2023.1082685

**Published:** 2023-02-09

**Authors:** Rasa Smaliukienė, Svajone Bekesiene, Rosita Kanapeckaitė, Olga Navickienė, Ieva Meidutė-Kavaliauskienė, Ramutė Vaičaitienė

**Affiliations:** General Jonas Zemaitis Military Academy of Lithuania, Vilnius, Lithuania

**Keywords:** prosocial motivation, meaning in work, meaning in military service, reserve soldiers, role fit, mediation, moderation

## Abstract

**Introduction:**

The meaningful commitment to serve one’s country and the desire to defend others make military service unique compared to other human activities. This is especially true for the army reservists who are typically working in the civilian labor market and serving military for the short-term military training or military missions only. As scholars provide limited insights into the effects and influences of prosocial motivation on meaning in military service, this study contributes to the understanding of direct, mediated, and moderated processes which link prosocial motivation to meaning in military service among reservists. Specifically, the objective of this study was to examine both direct and indirect pathways interconnecting prosocial motivation and meaning in military service. The former is analyzed as a direct effect, while the latter includes the effects of role fit within the military environment, the soldiers’ self-efficacy, as well as the socio-moral climate of military organization—that is, a variable making military service an exceptional activity.

**Methods:**

This study followed a quantitative method analysis by utilizing a hierarchical regression analysis which revealed direct, moderating, and mediating links between the variables. The analysis was based on a sample of 375 soldiers from the Active Reserve of the Lithuanian Armed Forces, and the data were collected before and after training exercises in one military unit (repeated measures). The effects on providing meaning to military service were evaluated using the following: Occupational Self-Efficacy Scale, Prosocial Motivation Scale, Motivation at Work Scale, and Socio-Moral Climate Scale. Prosocial motivation assumes meaning in military service among reservists through different, yet related, pathways.

**Results and discussion:**

The direct pathway confirmed that reserve soldiers with higher levels of prosocial motivation experience a higher level of meaning in service. The indirect pathway indicated that role fit mediated this relationship. Following the latter, we found that prosocial motivation was a significant predictor of both role fit and meaning in military service. Finally, we confirmed the moderated-mediation effects of self-efficacy and socio-moral climate in our suggested models. The results can be used to improve training programs for reservists.

## 1. Introduction

Reservists are civilians who are constantly moving between military and civilian fields (Laanepere and Kasearu, 2021), usually working in the civilian labor market (Figinski, 2017) and participating in short-term military training or military missions. In democracies, reservist service in active military reserve is mainly on a voluntary basis, so civilians have to find meaning in staying active in military reserve and updating constantly their military skills. For reservists, military service is an additional component in multiple life roles that can disturb (Harnett and DeSimone, 2011) or ail (Sweet and Black, 2021) work-life balance.

Over the last decades, the importance of the role of reservists in the army has drawn much academic attention. A large body of research in this field places a strong emphasis on personnel administration; that is, recruiting (Figinski, 2017) and retention (Perry et al., 1991). Few studies, however, have examined the tacit elements of reservist service. A set of tacit issues are found to have an impact on the military service of reservists in terms of their beliefs, attitudes, intentions, and, finally, citizenship or “service to the country” (Griffith and Ben-Ari, 2021). Personal motivation to being “committed to serving others” (McInnis, 2017) makes military service meaningful. Yet, a direct pathway between the intention to benefit others (prosocial motivation) and the meaning in military service is only part of the story, in that the latter is just the result of a set of subjectively perceived variables. For reservists, military service is often related with citizenship. In countries where military reservist service is compulsory, military readiness (training and other activities) is an inalienable part of civic duty. Reservists also need to maintain a link with their unit and their civilian habitus in order to be able to perform their reserve service successfully (Laanepere and Kasearu, 2021). This citizen role is an important element in military service by reservists, for these citizen-soldiers are constant transmigrants between their civilian and military life (Lomsky-Feder et al., 2007; Gazit et al., 2021). In the course of their duties in the army, reservists have to identify themselves with dissimilar roles than in civilian life, which can affect how one perceives meaning in military service. Military service, however, demands self-determination and personal character that help accomplish physically and psychologically demanding tasks during military training (Nindl et al., 2018). The military environment is also paramount as military organizations support their members through unique social and moral values (Kirke, 2010). All these variables which make military service different from civil work need to be analyzed in order to understand complex processes linking prosocial motivation to meaning in military service among reservists. In this case, prosocial motivation is understood as a willingness to benefit other soldiers in the unit to “produce and maintain the well-being and integrity of others” (Hannah et al., 2011, p. 556) and the society at large (Castanheira et al., 2016).

There has been much debate about prosocial motivation within the military (Lebel, 2012; Castanheira et al., 2016; Griffith and Ben-Ari, 2021), but only a few studies have examined their relationship with the meaning in military service. Most of the relationships were found by chance, as they were included in studies that looked at military leadership variables (see Gunn, 2016; Sefidan et al., 2021; Salem et al., 2022), for example, it is found that prosocial motivation in the military is stimulated by a supportive socio-moral climate created by a unit leader (Lebel, 2012); role modeling is concluded to positively influence prosocial motivation in the military (Kim and Vandenberghe, 2020). Additionally, there is a large body of research emphasizing the effect of self-efficacy on meaning-making; self-efficacy is “part of a necessary meaning-making process” (Narayanan and Ordynans, 2022, p. 25). This is particularly true in the military where self-efficacy and other personal qualities become important in the challenging military context. Yet, there is a paucity of scientific resources examining the impact of self-efficacy on meaning in military service by reservists who are both civilians and soldiers, as discussed above.

Research has shown that in addition to the direct prosocial motivational effect in the military, various contextual influences can affect the meaning of motivation. Therefore, the objective of this study was to examine the direct and indirect pathways linking two phenomena – prosocial motivation and meaning in military service. We have extended our analysis to non-direct relationships by incorporating other variables which make military service a specific activity and run as follows: effects of role fit within the military environment, soldiers’ self-efficacy, and socio-moral climate of military organization. This research was carried out in Lithuania where active military reserve is comprised of conscripts who attained basic military training no later than 10 years ago. Thorough these years, reservists are to participate in exercises and training for 20–60 days and are called to update their military skills every few years. This format of military service enables reservists to integrate military service into their civilian life, but at the same time, it becomes problematic to maintain the focus and priority of military service. All this poses a question of whether prosocial motivation—the desire to protect and serve others—helps make military service meaningful to reservists. We conducted the study twice, before and after the training of reservists, in order to assess changes in the impact of social motivation on meaning in military service.

## 2. Measuring meaning in military service by reservists: Hypothesis development

### 2.1. The role of prosocial behavior in meaning-making of military service

Meaning-making in the military is associated with the activities person performs. According to the classical description provided by Pratt and Ashforth (2003, p. 311), meaning of work is a process of sense-making when “the work and/or its context are perceived by its practitioners to be, at a minimum, purposeful and significant.” Research shows that this meaning is a dimension that lies beyond the traditional models of organization and organization management (Schnell et al., 2013). Here, the discussion includes the various constructs of meaning that relate to a humanistic approach to human motivation and the search for meaning. Following Rogers’ (1977) classical humanist approach, Willmott et al. (2018) argue that people have a natural need for positive regard, which leads to the formation of a positive self-concept. So, meaning of work is a deep positive feeling that is related to interpretation of the real self.

In the military context, it is about transcending own self-interest to pursue the greater good. Applying the definition by Pratt and Ashforth (2003, p. 311), meaning in military service is a subjective sense when soldiers perceive their activities in the military domain as purposeful and significant to the society. Members of military organizations usually speak of a vocation and express their activities in terms such as “duty” and “honor,” which distinguish them from the general public (Moskos, 2005).

According to Grant (2007), activities wherein a person can have an impact on the beneficiaries of his or her activity (e.g., colleagues, clients, victims, patients, and civilians) increase a perceived social value and are more meaningful to the individual. In the milieu of military service, scientific resources suggest that the rationale for serving in the military is associated with a number of intrinsic and extrinsic factors (Taylor et al., 2015) and relates not only to pragmatic gains (as pay and benefits) and self-centered benefits (as self-development, job skills or adventure) but also to altruistic motives expressed in terms of duty and patriotism (Woodruff et al., 2006). In the reservist service, the notion of meaning is associated with altruistic reasons like “acquire values” (Masson, 2022), serve one’s country, and be more useful to one’s community (Griffith and Ben-Ari, 2021).

Therefore, it can be assumed that military service for reservists is characterized by the desire to exert a positive impact on other people; that is by prosocial motivation. Taking into account the social aspects of their tasks, we expected that meaning in military service was reinforced by prosocial motivation when military reservists perceive their impact on other people. And hence, we hypothesize:

*H1:* Prosocial motivation has a positive effect on the meaning of military service. As such, reserve soldiers with higher levels of prosocial motivation tend to perceive a higher level of meaning in military service.

### 2.2. The role of role fit, self-efficacy, and organizational support in meaning-making of military service

The meaning in military service is affected by individual and contextual variables. It is a tacit dimension that depends on the personal sources of meaning and on the role the person is playing in an organization and on the organization itself (Schnell et al., 2013). Considering the meaning in military service as a complex subjective construct, what becomes pivotal is direct and indirect pathways linking it to prosocial motivation. Steger et al. (2012) state that it is not enough to use the task characteristics as proxies to evaluate if military service is meaningful; experiential dimensions need to be included, too. This particularly applies to the context of military reservists since their military service is a duty of citizens. Three variables need to be considered in this context.

First, the dual duty of citizens and solders raises the issue of *role fit* within military organizations (Moskos, 2005). Ideally, the role should be in line with the person’s identity and purpose in life, considering present and future goals. Only then will a person be able to express his or her values, beliefs, and talents and feel intrinsically motivated (Schnell et al., 2013). Additionally, in the scholarly literature on civic engagement, it is revealed that serving others (prosocial behavior) increases role fit (Homberg and Costello, 2019). Consequently, role fit must be included in an indirect pathway linking prosocial motivation and meaning in military service.

Second, the soldier’s personality measures are found to be critical in meaning-making, for they determine how soldiers approach a stressful or challenging situation (Taylor et al., 2013) and how they perceive themselves in a given situation (Rumsey, 2020). More specific, perceived self-efficacy is concerned with a soldier’s beliefs in his/her capabilities to control and make an impact (Bandura, 1999). The soldier’s self-efficacy has been theoretically and empirically linked to increased resilience (Djourova et al., 2020; Bekesiene et al., 2022) and positive adaptation in day-to-day life (Butler et al., 2021). As described by Schnell et al. (2013), self-efficacy allows a person to perceive “one’s acts as significant and therefore meaningful.”

Third, following research, meaning is formed by not only personal but also organizational-level variables such as structure and culture (Sun et al., 2019). In the military context, organizational-level variables are related to the *socio-moral climate* within a military organization (Krämer-Badoni and Wakenhut, 2016). It should be noted that research reveals a significant effect of socio-moral contexts in military organizations, ranging from highly motivating such as cohesion and esprit de corps (Gal and Mangelsdorff, 1991) to undermining ones related to trauma, threats to personal safety violence (van Voorhees et al., 2018). Studies show that camaraderie and mutual support make military service a subjectively meaningful activity (Roth, 2021; Smaliukienė et al., 2022).

**Figure 1:**
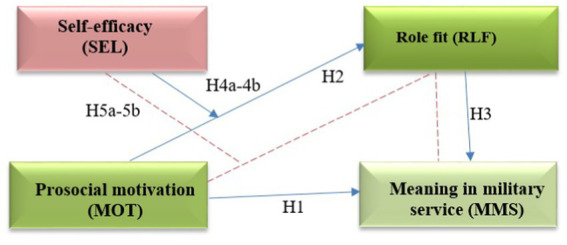
Hypothesized theoretical model (at the beginning of military training): H1 and H2 represent main effect of prosocial motivation (MOT), H3—the mediation of Role fit (RLF), H4a and H4b—the moderation effects, and H5a and H5b—the indirect effect of prosocial motivation (MOT) on Meaning in military service (MMS) through Role fit (RLF).

**Figure 2:**
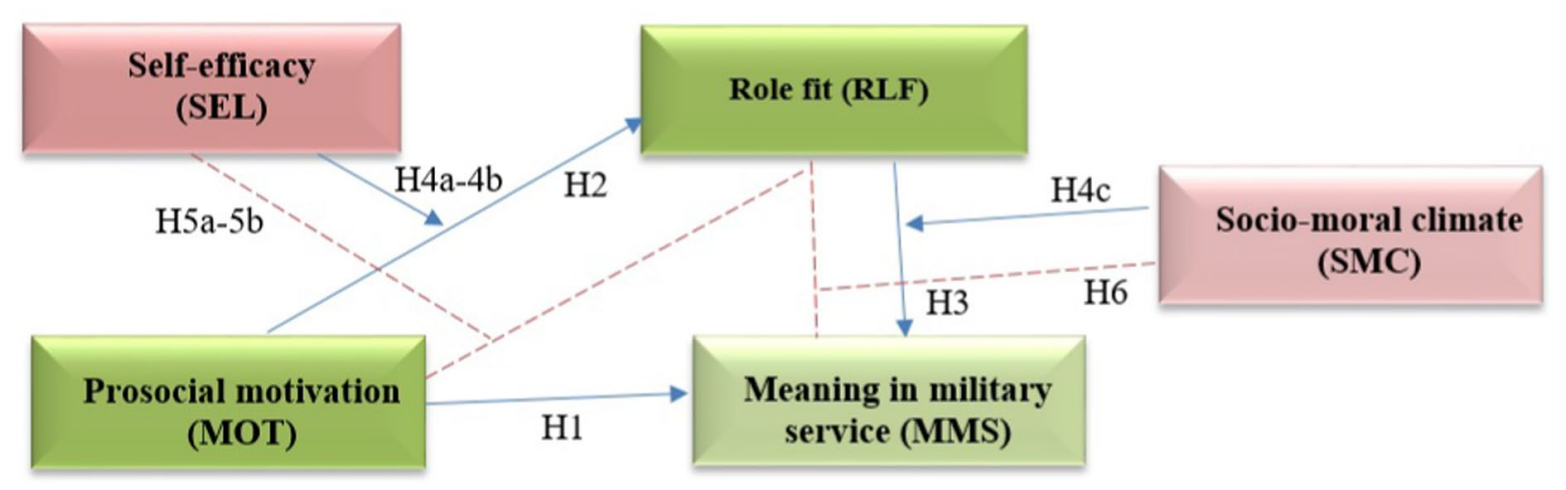
Hypothesized theoretical moderated-mediation model (at the end of military training): H1 and H2 represent main effect of prosocial motivation (MOT2), H3—the mediation of Role fit (RLF), H4a, H4b, and H4c—the moderation effects, and H5—the indirect effect of prosocial motivation (MOT2) on meaning in military service (MMS) through Role fit (RLF).

Considering all the above mentioned, the meaning in reservist military service is associated not only with prosocial motivation but also with role fit, self-efficacy, and organizational support. Given the importance of prosocial motivation in the service of reservists, the other three measures should be considered to have an indirect (corrective) effect on the meaning in military service. To combine all the variables into a hypothetical model, we refer to the guidelines for the moderated-mediation model in psychology by Muller et al. (2005). According to the guidelines, the mediator is responsible for a causal effect between variables. In the reservist service, it is important how effectively one reconciles one’s duties both in civilian and military lives, so role fit could serve as a mediator in understanding the effect of prosocial motivation on the meaning in military service. Following the guidelines, the moderator is either an individual difference variable or a contextual variable (Muller et al., 2005). In our case, self-efficacy serves as the former, while socio-moral climate in a military unit serves as the latter. We thus hypothesize links in direct and indirect pathways from prosocial behavior to meaning in military service by reservists (see Figures 1, 2).

Direct effect:

*H2:* Prosocial motivation has a positive effect on role fit, so that reserve soldiers with higher levels of prosocial motivation tend to have higher levels of role fit compared to those with lower levels of prosocial motivation.

Mediation effect:

*H3:* Role fit positively mediates the relationship between prosocial motivation and meaning in military service such that reserve soldiers with higher levels of role fit tend to perceive a higher level of meaning in military service in comparison to those with lower levels of role fit.

Moderation effects:

*H4a:* Self-efficacy positively moderates the relationship between role fit and meaning in military service such that the positive effect of self-efficacy on the meaning in military service is enhanced for reserve soldiers with high self-efficacy and mitigated for reservists with low self-efficacy.

*H4b:* Self-efficacy positively moderates the relationship between prosocial motivation and meaning in military service such that the effects of prosocial motivation on meaningful service are enhanced for soldiers with high self-efficacy and mitigated for soldiers with low self-efficacy.

*H4c:* Socio-moral climate positively moderates the relationship between role fit and meaning in military service such that the effect of role fit on the meaning in military service is enhanced for reserve soldiers with high self-efficacy and mitigated for soldiers with low socio-moral climate.

Should the above hypotheses be proven, moderated mediation will be analyzed further. According to the guidelines, moderated mediation happens only if mediating occurs (Muller et al., 2005). The hypothesis for moderated-mediation effects is as follows:

*H5:* Self-efficacy positively moderates the strength of the mediated relationship between prosocial motivation and meaning in military service through role fit, so that the mediated relationship is stronger under high self-efficacy than under low self-efficacy.

*H6:* Socio-moral climate positively moderates the strength of the mediated relationship between prosocial motivation and meaning in military service through role fit such that the mediated relationship is stronger under a high socio-moral climate than under a low socio-moral climate.

## 3. Methods

### 3.1. Participants and procedures

Data were collected from 375 soldiers from the Active Reserve (AR) of the Lithuanian Armed Forces (LAFS), using a self-administrated questionnaire twice: at the beginning (BMTM, 1st time point) and at the end of training (EMTM, 2nd time point). Three hundred fifty-three valid questionnaires were used to analyze 1st time point, and 330 questionnaires—for the 2nd time point.

The sample included all soldiers who participated in a 5-week reserve training course. This platoon-level field tactical exercise focuses on updating individual soldiering skills and expanding teamwork experience. Additionally, it can be mentioned that all participants completed the mandatory tasks and operations under the supervision of professional LAFS instructors. During this training, all participants remained in the same training area (without going home) and spent 5 weeks in the barracks.

The average age of soldiers was 28.5 years (SD = ± 1.29, range = 21–40 years). All study participants were men; this happens as military service in Lithuania is only compulsory for men. The majority were specialists in the labor market 209 (59%); the minority were managers who had subordinates 46 (13%); and the others 99 (28%) held different positions. The respondents mostly represent the private sector – 259 (73%). Furthermore, 34% of the respondents had experience, because they had previously been trained in different types of military training.

The study was carried out over two time points. At the beginning of training (1st time point), the respondents received a questionnaire containing Self-Efficacy Scale, Prosocial Motivation Scale, Motivation at Work Scale, and Meaning in Military Service Scale. At the end of training (2nd time point), the respondents received the same questionnaire supplemented by the Socio-Moral Climate Scale to evaluate the organizational support during the training. To coordinate data across these two time points, respondents were coded.

### 3.2. Measures

#### 3.2.1. Self-efficacy scale

Self-efficacy was measured using an occupational self-efficacy scale (OCCSEFF). The original version was developed by Schyns and von Collani (2002) and adopted in the military training environment by Buch et al. (2015). The 7-item version by Buch et al. (2015) measures self-efficacy expectations in the military domain. For each item, “With reasonable certainty, I can say that I” is used to measure perceived military competences. We used Buch et al. (2015) statements at the beginning and end of military training. Sample items included “…will achieve results that I can be proud of”; some of the statements we had to adapt to the AAPR case: “…will be able to endure the most difficult moments of AAPR.” Reserve soldiers rated items on a 5-point scale from 1 point (strongly disagree) to 5 points (completely agree). The Buch et al. (2015) scale had good internal consistency (Cronbach’s *α* of 0.89), as well as the present study [Cronbach’s *α* 0.884 (1st time point) and 0.922 (2nd time point)].

#### 3.2.2. Prosocial motivation scale

Prosocial motivation was measured using Grant’s (2008) four-item prosocial motivation scale. The Prosocial Motivation Scale measures the willingness to make an effort to help other people or contribute to their well-being. The scale was used in a similar study at a military base (Grant and Berry, 2011) and sowed high internal consistency (Cronbach’s *α* 0.91). We adapted the statements to the training context (here is a sample item: “I care about benefiting others through my participation in these trainings”). The responses were evaluated on a Likert scale from 1 point (strongly disagree) to 5 points (completely agree). The scale had good internal consistency in this study, Cronbach’s *α* 0.880 (1st time point) and 0.886 (2nd time point).

#### 3.2.3. Motivation at work scale

Role fit within the reservist service was measured using Motivation at Work Scale (Gagné et al., 2010). Gagné et al. (2010) created items to measure different work-related behavioral regulations that represent the range of the continuum of motivation to do a particular job by including instinct motivation for doing that. The two measures on this scale represent what is described as a roll-fit: (i) Identified Regulation on this scale implies that the person performing an activity identifies with its value to the extent that it becomes part of a person’s routine and self-perception; (ii) Intrinsic Motivation is defined as doing something that is interesting and enjoyable. These measures are tested in the various contexts, including the military. In the military context, it was found that intrinsic motivation reflects identification with military values (Sáiz-Pardo et al., 2021). Overall, the measures showed good internal consistency in the previous studies as well as in our study [Cronbach’s *α* 0.900 (1st time point) and 0.928 (2nd time point)]. We applied the measures of identified regulation and intrinsic motivation by modifying the statements according to the military context (here is a sample item: “Because it’s fun serving at military”). The answers were evaluated on a Likert scale from 1 point (strongly disagree) to 5 points (completely agree).

#### 3.2.4. Meaning in military service scale

According to the self-determination theory and the findings on the meaning in military service in the previous studies, three levels of meaning were investigated: (i) military service as a morally inspiring act, (ii) meaningful performance of duty, and (iii) internal satisfaction in performing military service. Our scale is based on the Work and Meaning Inventory (WAMI; Steger et al., 2012), which consists of three dimensions: greater good motivations; meaning-making through work; and positive meaning. The inventory is used extensively as WAMI is just one of only several meaning-at-work inventories used in empirical studies (Puchalska-Kamińska et al., 2019). The three-item scale was developed for this study. The statements are worded in a way that are relevant to the military service by reservists (here is a sample item: “Because I see opportunities to fulfill myself as a Lithuanian citizen”). The answers are evaluated on a Likert scale from 1 point (strongly disagree) to 5 points (completely agree); the scale had good internal consistency, Cronbach’s *α* 0.837 (1st time point) and 0.873 (2nd time point).

#### 3.2.5. Socio-moral climate scale

Socio-moral climate was measured using a three-item scale that measures organizational support, supervisor support, and camaraderie. Organizational support was measured as a concern for the well-being of soldiers; supervisor support dealt with the ability to facilitate effective interactions between soldiers; and camaraderie contribution reflected the extent to which it is perceived as supportive. These three statements were adopted from Krueger et al. (2002) and were found to be indispensable to the formation of a socio-moral climate in the military (Greenberg and Jones, 2010; here goes a sample item: “The support of colleagues played an important role”). The answers were evaluated on a Likert scale from 1 point (strongly disagree) to 5 points (completely agree) and showed acceptable internal consistency Cronbach’s *α* 0.783.

### 3.3. Methods of statistical analysis

The G∗Power v3.1.9.4 test was conducted for the sample size evaluation of collected data prior to the statistical analyses for the 1st and 2nd time points. The statistical hypothesis testing was based on three predictors that also accounted for the moderator with a significance level of 0.05, power of 0.95, and effect size of 0.15. It was indicated that a minimum sample size of 119 is required to reach statistical power. Additionally, the post-hoc test was used to compute achieved power. It was revealed that even with a sample size of 349 it can reach statistical power equal to 0.999; in our study, we used a sample size of 353 (valid questionnaires were used to analyze 1st time point). Also, 330 valid questionnaires were used for the 2nd time point, while the post-hoc analysis showed that even with a sample size of 326 and effect size of 0.15 it can reach statistical power equal to 0.999. To examine the hypothetical models, the modeling analyses were performed by using IBM SPSS Statistics 28v and SPSS AMOS 28v. First, demographic data were computed at the individual level of analysis, and primary data analysis along with our study foregrounds prosocial motivation (MOT), self-efficacy (SEL), role fit (RLF), and socio-moral climate (SMC) on the meaning in military service (MMS) of reserve soldiers. Descriptive statistics (i.e., *M*, ± SD) were calculated for the study variables, and the relationships between constructs were evaluated using Pearson’s bivariate correlation. The strength of relationships was reported following Cohen’s (2013) recommendations. Furthermore, to continue these investigations, the differences in value between the 1st and the 2nd time points measures were tested by the paired-sample *t*-test.

Second, Harman’s single-factor test was used to investigate the potential variance of the common method (Tehseen et al., 2017). The average variance extracted (AVE) was measured to show the construct convergence (Fornell and Larcker, 1981). In addition, confirmatory factor analyzes were performed in AMOS 28v to establish the psychometric properties of the study scales. Furthermore, discriminant validity and confidence intervals were chosen to disclose evidence of discriminant validity between constructs (Tehseen et al., 2017). The goodness-of-fit of the structural regression models was evaluated by means of a comparative fit index (CFI) and Tucker-Lewis Index (TLI) with values in the area of 0.90–0.95, as indicators of good fit (Maydeu-Olivares and García-Forero, 2010). The model fit is also reported using the following: a *χ*^2^ (df), a Comparative Fit Index (CFI) >0.90; and a root mean square error of approximation of the study’s methodology is presented.

Third, the hypothesized moderated-mediation models for two time point measures were investigated. To confirm a mediation effect in hypothesized models, we used a four-step data analysis procedure offered by Fritz et al. (2012). The assessment and identification analysis procedures were performed using Hayes’s PROCESS v3.5 macro models (Model 4, and Model 7) and included: (i) a significant relationship between prosocial motivation (MOT) and meaning in military service (MMS) of reserve soldiers, (ii) a significant association between prosocial motivation (MOT) and role fit (RLF), (iii) a significant relationship between role fit (RLF) and meaning in military service (MMS) after controlling for prosocial motivation (MOT), and (iv) a significant coefficient for the indirect path between prosocial motivation (MOT) and meaning in military service (MMS) through self-efficacy (SEL) PROCESS v3.5 macro-Model 7 (Preacher and Hayes, 2004). The bootstrapping analysis of 5,000 was conducted, and the trust section was set at 95% bias-corrected CI. Furthermore, the moderated-mediation analysis was performed using Hayes’s PROCESS v3.5 macro-Model 59 (Hayes and Scharkow, 2013), and the degree of a mediation effect was observed on the value of a moderator (Muller et al., 2005). The indirect effect was considered statistically significant if the 95% bias-corrected CI did not include zero (Hayes and Scharkow, 2013).

## 4. Study results

### 4.1. Preliminary analyses for scale evaluations

Descriptive information on research variables is presented in Table 1.

**Table 1:** The descriptive statistics, discriminant validity, and Pearson’s correlations between the study variables.

Factor	Descriptive		Discriminant Validity		Correlations				
	*M*	±SD	CR	AVE	F1	F2	F3	F4	F5
**At the beginning**									
F1: Self-efficacy	4.414	0.645	0.916	0.646	**0.804**				
F2: Prosocial motivation	3.703	0.889	0.932	0.774	0.507**	**0.880**			
F3: Role fit	3.574	0.847	0.923	0.669	0.574**	0.665**	**0.818**		
F4: Meaning in military service	3.375	0.934	0.903	0.757	0.491**	0.615**	0.813**	**0.870**	**–**
**At the end**									
F1: Self-efficacy	4.364	0.767	0.941	0.728	**0.853**				
F2: Prosocial motivation	3.780	0.895	0.935	0.783	0.591**	**0.885**			
F3: Role fit	3.400	0.985	0.943	0.737	0.598**	0.695**	**0.858**		
F4: Meaning in military service	3.274	1.019	0.922	0.799	0.546**	0.705**	0.900**	**0.894**	
F5: Socio-moral climate	3.881	0.755	0.836	0.630	0.511**	0.532**	0.530**	0.521**	**0.794**

F1 = capability to harness the circumstances [Self-efficacy (SEL)]; F2 = Prosocial Motivation (MOT); F3 = Role fit (RLF); F4 = Meaning in military service; F5 = Socio-moral climate (SMC). **Pearson’s correlation is significant at the 0.01 level (2-tailed). *M*, means; ±SD, standard deviations. Bold values on diagonal are the square root of AVE for each construct.

The correlation analysis for the data collected at the 1st time point showed that prosocial motivation was positively associated with meaning in military service (MOT&MMS, *r* = 0.615, *p* < 0.01), role fit (MOT&RLF, *r* = 0.665, *p* < 0.01), and self-efficacy (MOT&SEL, *r* = 0.507, *p* < 0.01). Self-efficacy was strongly positively associated with role fit (SEL&RLF, *r* = 0.507, *p* < 0.01). Finally, role fit was highly positively related to meaning in military service (RLF&*MMS, r* = 0.870, *p* < 0.01).

Harman’s single-factor test was conducted. The findings of exploratory factor analysis (EFA) accounted for 66.52% (BMTM) and 72.29%(EMTM) of the total variance for greater than one factor, and 32.54% (BMTM) and 39.37% (EMTM) of the covariance between the measures was indicated for a single factor. This analysis proved that there are no problems associated with the common-method variance in data. Additionally, all assessed constructs demonstrated adequate reliability, with composite reliability (CR) coefficients ranging from 0.903 to 0.932. Discriminant validity was also supported, as the extracted average variance (AVE) values ranged from 0.646 to 0.774 according to the requirements (see Fornell and Larcker, 1981). Moreover, the common-method bias was assessed with a single latent factor using the full-scale items as indicators and did not show highly correlated variables.

The analysis of data from the 2nd time point revealed that prosocial motivation was also highly significant and positively associated with meaning in military service (MOT&MMS, *r* = 0.705, *p* < 0.01) as well as with role fit (MOT&RLF, *r* = 0.695, *p* < 0.01). Moreover, role fit was highly positively related to meaning in military service (RLF&MMS, *r* = 0.900, *p* < 0.01). Additionally, composite reliability (CR) was tested, and it ranged from 0.838 to 0.943, and AVE values ranged from 0.630 to 0.799 in accordance with the requirements (see Fornell and Larcker, 1981). Furthermore, the single latent factor using full-scale items as indicators did not show highly correlated variables.

The data validity analysis results let us continue with the SEM analysis, which was performed by IBM AMOS 28v. Confirmatory factor analysis procedures were used to test the validity of the overall model constructs, as theorized in two analysis models: BMTM (at the beginning of military training) and EMTM (at the end of military training). The computed SEM analyzes on hypothesized models let us identify the goodness-of-fit of constructs (see Table 2).

**Table 2:** Hypothesized models evaluation by goodness-of-fit statistics.

Index fit	Recommended value^1^	Theorized models	
		BMTM	EMTM
CMIN/DF	≤3	0.578	1.367
Probability level	>0.05	0.447	0.255
RMSEA	≤0.08	0.000	0.032
NFI	≥0.9	0.999	0.998
CFI	≥0.9	1.000	0.999
TLI	≥0.9	1.003	0.995
AIC	The lower is better	18.578	38.734
BCC	The lower is better	18.837	39.356

1 Hair et al. (2010) recommended value.

The goodness-of-fit of the constructed theoretical models was appropriate, that is, better (lower) than the standard score of three suggested by scholars (Bekešienė et al., 2017): *χ*^2^/df = 0.578 for BMTM and *χ*^2^/df = 1.367 for EMTM. The root mean square error of approximation (RMSEA) had scores lower than the maximum allowed (Hair, 2019), for BMTM, it was 0.000, and for EMTM, 0.032. All the scores of the normed-fit index (BMTM: NFI = 0.999; EMTM: NFI = 0.998), competitive fit index (BMTM: CFI = 1.000; EMTM: CFI = 0.999) exceed 0.9 as recommended (Bekešienė et al., 2017; Bekesiene and Hoskova-Mayerova, 2018), and the Tucker-Lewis Index (TLI) has scores (BMTM: TLI = 1.003; EMTM: TLI = 0.995) that matches the recommendations (Hair, 2019). The goodness-of-fit results indicate that the designed measurement models exceeded the minimum values (Table 2).

Furthermore, the differences in value between the study variables were repeatedly tested. The paired samples *t*-test confirmed the significant differences for self-efficacy (SEL, ∆BT vs. AT) = 0.077, *p* < 0.05), role fit (RLF, ∆BT vs. AT) = 0.202, *p* < 0.01), and meaning in military service (MMS, ∆BT vs. AT) = 0.119, *p* < 0.05). Prosocial motivation values did not change statistically significant (MOT, ∆BT vs. AT) = −0.043, *p* > 0.05), see Table 3.

**Table 3:** Paired differences between study variables identified by paired samples *t*-test.

Paired samples	Paired differences		Difference by 95% CI		*t* statistic	df	Sig.
	Mean change	SD	Lower	Upper			
Pair 1: Self-efficacy (BT) vs. (AT)	0.077	0.700	0.001	0.152	10.99	330	0.047
Pair 2: Prosocial Motivation (BT) vs. (AT)	−0.043	0.858	−0.136	0.050	– 0.913	330	0.362
Pair 3: Role fit (BT) vs. (AT)	0.202	0.828	0.113	0.300	40.449	330	0.000
Pair 4: Meaning in military service (BT) vs. (AT)	0.119	0.924	0.019	0.219	20.339	330	0.020

BT, before training; AT, after training.

### 4.2. Hypotheses testing results

#### 4.2.1. The direct effects

Direct effects (i.e., H1 and H2) in the BMTM and EMTM models were tested using the PROCESS v3.5 macros simple mediation model construction (Model 4) as suggested by scholars (Hayes, 2022). The average values of the total score of items represented the constructs. So, prosocial motivation was found to have a significant, positive, and direct influence on meaning in military service (BMTM, MOT → MMS for H1: *β* = 0.690, *p* = 0.000; EMTM, MOT → MMS for H1: *β* = 0.804, *p* = 0.000), and role fit (RLF) (BMTM, MOT → RLF for H2: *β* = 0.673, *p* = 0.000; EMTM, MOT → RLF for H1: *β* = 0.765, *p* = 0.000). Consequently, H1 and H2 are accepted for both models (see Table 4). As such, reserve soldiers with higher levels of prosocial motivation tend to perceive a higher level of meaning of military service (H1), and reserve soldiers with higher levels of prosocial motivation tend to have higher levels of role fit compared to those with lower levels of prosocial motivation (H2).

**Table 4:** The direct effects of Prosocial Motivation (MOT) evaluated by using the PROCESS v3.5 macro-Model 4.

	Explanation			Coeff. *β*	SE	St. Coeff. *β*	*t*	Value of *p*	LLCI	ULCI
At the beginning of training (BMTM)	Hypothesis H1	**Model 1 (H1)**	Constant	0.818	0.160		5.099	0.000	0.503	1.134
		MOT → MMS	MOT	0.690	0.042	0.658	16.384	0.000	0.607	0.773
		**Model 1** **Summary**	***R***	***R*-sq**	**MSE**	***F***	**df1**	**df2**	**Value of *p***	
			0.658	0.433	0.496	268.435	1.000	352.000	0.000	
				**Coeff. *β***	**SE**	**St. Coeff. *β***	***t***	**Value of *p***	**LLCI**	**ULCI**
	Hypothesis H2	**Model 2 (H2)** MOT → RLF	Constant	1.080	0.137		7.907	0.002	0.811	1.348
			MOT	0.673	0.036	0.707	18.781	0.000	0.603	0.744
		**Model 2** **Summary**	***R***	***R*-sq**	**MSE**	***F***	**df1**	**df2**	**Value of *p***	
			0.707	0.501	0.359	352.715	1.000	352.000	0.000	
At the end of training (EMTM)	Hypothesis H1	**Model 1 (H1)**	Constant	0.266	0.173		1.364	0.173	−0.104	0.576
		MOT → MMS	MOT	0.804	0.045	0.705	18.054	0.000	0.716	0.891
		**Model 1 Summary**	***R***	***R*-sq**	**MSE**	***F***	**df1**	**df2**	**Value of *p***	
			0.705	0.498	0.524	325.957	1.000	329.000	0.000	
				**Coeff. *β***	**SE**	**St. Coeff. *β***	***t***	**Value of *p***	**LLCI**	**ULCI**
	Hypothesis H2	**Model 2 (H2)** MOT → RLF	Constant	0.506	0.170		2.986	0.003	0. 173	0.840
			MOT	0.765	0.044	0.695	17.529	0.000	0.680	0.851
		**Model 2 Summary**	***R***	***R*-sq**	**MSE**	***F***	**df1**	**df2**	**Value of *p***	
			0.695	0.483	0.504	307.262	1.000	329.000	0.000	

Model 1 = outcome variable MMS (Meaning in military service); Model 2 = outcome variable RLF (Role fit); *R* = correlation coefficient and *R*-sq = correlation coefficient in square. LLCI = lower bound of 95% CI; ULCI = upper bound of 95% CI. Bootstrap sample size = 5,000.

#### 4.2.2. Testing for mediation effect

In Hypothesis 3, we predicted that role fit (RLF) positively mediates the relationship between prosocial motivation (MOT) and meaning in military service (MMS). Hypothesis testing was conducted in a four-step procedure separately for two theorized models (BMTM and EMTM). Additionally, the bias-corrected percentile bootstrap approach was used to determine whether the conditions were fulfilled.

The indirect effect of prosocial motivation on meaning in military service *via* role fit (MOT → RLF → MMS) was based on 5,000 bootstrap samples estimated with a 95% CI as suggested by (Hayes, 2022) and was investigated in both models (BMTM and EMTM) separately. The study results revealed that prosocial motivation was a significant predictor of role fit (BMTM model, MOT → RLF, *β* = 0.673, *p* = 0.000; EMTM model, MOT → RLF, *β* = 0.765, *p* = 0.000), and role fit was a significant predictor of meaning in military service (BMTM model, RLF → MMS for H3: *β* = 0.835, *p* = 0.000; EMTM model, RLF → MMS for H3: *β* = 0.820, *p* = 0.000). The study results show that ~72% (BMTM model, *R*^2^ = 0.719) and 82% (EMTM model, *R*^2^ = 0.823) of the variance in meaning in military service was accounted for by predictors of prosocial motivation (MOT) and role fit (RLF). Furthermore, we indicated that the indirect coefficient of prosocial motivation in meaning in military service was significant (BMTM model, *β* = 0.562, SE = 0.044, 95% CI = 0.476–0.652; EMTM model, *β* = 0.628, SE = 0.047, 95% CI = 0.539–0.724). Bootstrap with the 5,000 sample size test proved that all indirect effects were significant at *p* < 0.05 (Preacher and Hayes, 2004); no zero was included in the 95% CI in the BMTM model. Therefore, H3 is supported for both BMTM and EMTM models (see Table 5). As such, role fit positively mediates the relationship between prosocial motivation and meaning in military service such that reserve soldiers with higher levels of role fit tend to perceive a higher level of meaning of military service compared to those with lower levels of role fit.

**Table 5:** The mediation effects of role fit evaluated by using the PROCESS v3.5 macro-Model 4.

	Explanation			Coeff. *β*	SE	St. Coeff. *β*	*t*	Value of *p*	LLCI	ULCI
At the beginning of training (BMTM)	Hypothesis H3	**Model 3 (H3)**	Constant	−0.083	0.123		−0.677	0.499	−0.325	0.158
		MOT → MMS	MOT	0.128	0.042	0.122	3.051	0.002	0.046	0.211
		RLF → MMS	RLF	0.835	0.044	0.757	18.911	0.000	0.748	0.922
		**Indirect effect of prosocial motivation on meaning in military service**								
			**Effect**	**BootSE**	**Boot LLCI**	**BootULCI**			
		MOT → RLF → MMS		0.562	0.044	0.476	0.652			
		**Model 3 Summary**	***R***	***R*-sq**	**MSE**	***F***	**df1**	**df2**	**Value of *p***	
			0.848	0.719	0.246	449.013	2.000	351.000	0.000	
At the end of training (EMTM)	Hypothesis H3	**Model 3 (H3)**	Constant	−0.179	0.104		−1.722	0.086	−0.384	0.026
		MOT → MMS	MOT	0.176	0.037	0.154	4.777	0.000	0.103	0.248
		RLF → MMS	RLF	0.820	0.033	0.793	24.537	0.000	0.755	0.886
		**Indirect effect of prosocial motivation on meaning in military service**								
			**Effect**	**BootSE**	**Boot LLCI**	**BootULCI**			
		MOT → RLF → MMS		0.628	0.047	0.539	0.724			
		**Model 3 Summary**	***R***	***R*-sq**	**MSE**	***F***	**df1**	**df2**	**Value of *p***	
			0.907	0.823	0.185	761.762	2.000	328.000	0.000	

Model 3 = outcome variable MMS (Meaning in military service); Model 4 = outcome variable RLF (Role fit); *R* = correlation coefficient and *R*-sq = correlation coefficient in square. LLCI = lower bound of 95% CI; ULCI = upper bound of 95% CI. Testing for Moderation Effect. Bootstrap sample size = 5,000.

#### 4.2.3. Moderation effects

**Figure 3:**
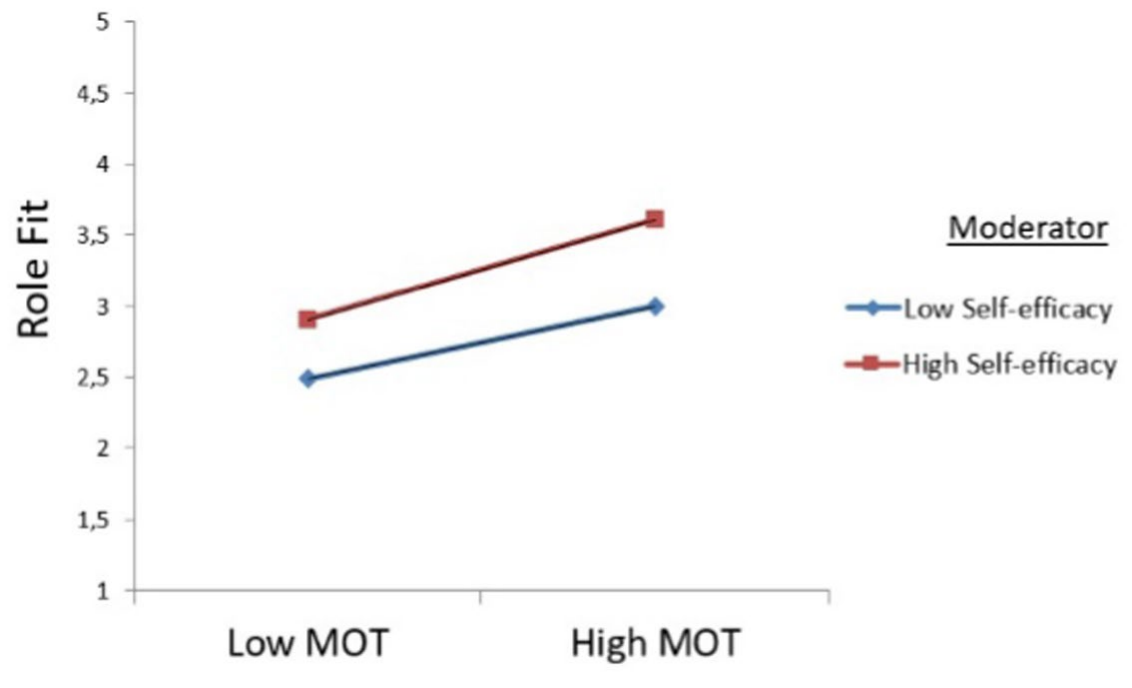
Graphical visualization of interactive effects for prosocial motivation (MOT) and self-efficacy (SEL) on role fit (RLF) at the beginning of military training (BMTM).

**Figure 4:**
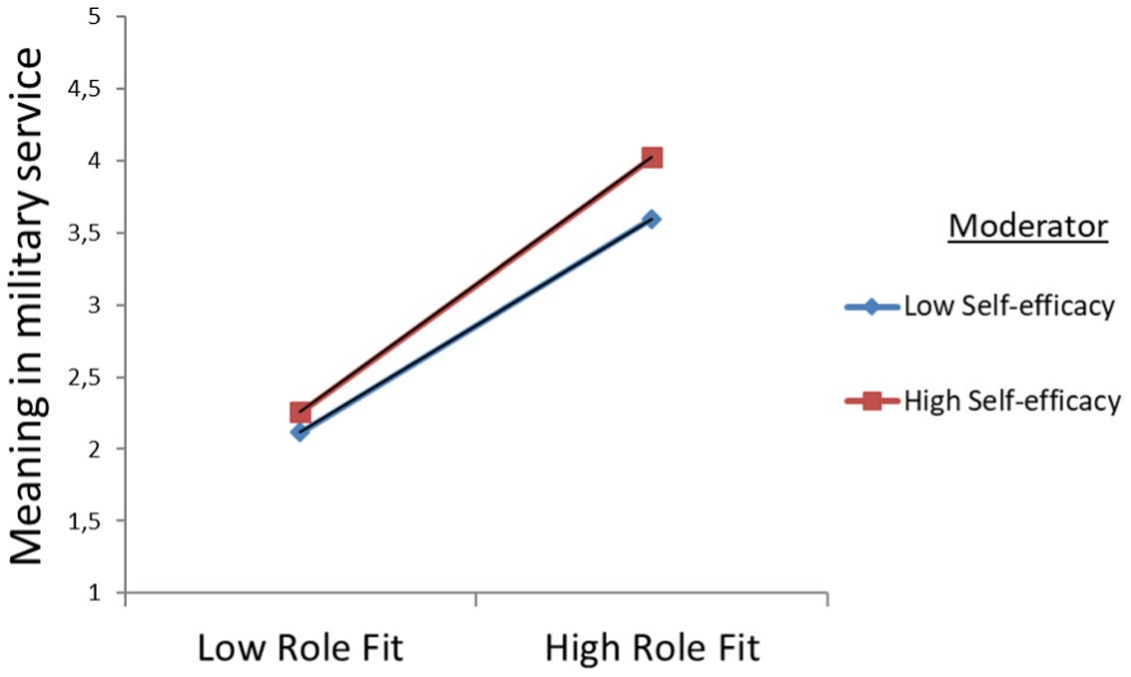
Graphical visualization of interactive effects for role fit (RLF) and self-efficacy (SEL) on meaning in military service (MMS) at the beginning of military training (BMTM).

The results of the moderation modeling conducted for BMTM demonstrated that role fit was associated with meaning in military service (BMTM, *β* = 0.813, *p* = 0.009). Furthermore, the moderating effect of self-efficacy on the relationship between role fit and prosocial motivation showed significant positive interaction effects, with (1) prosocial motivation and self-efficacy in role fit (BMTM, MOT × SEL, *β* = 0.048, *p* = 0.022), and (2) role fit and self-efficacy in meaning in military service (BMTM, RLF × SEL, *β* = 0.007, *p* = 0.048). The results for BMTM are presented by simple slope graphs [low (−1 SD) vs. high (+1 SD) levels of self-efficacy] that unfolded the association between prosocial motivation and role fit. The modeling let us point out a significantly positive association between self-efficacy and prosocial motivation, as well as between self-efficacy and role fit. Therefore, it can be concluded that self-efficacy strengthens the positive relationship between MOT and RLF (see Figure 3), and between RLF and MMS (see Figure 4).

**Figure 5:**
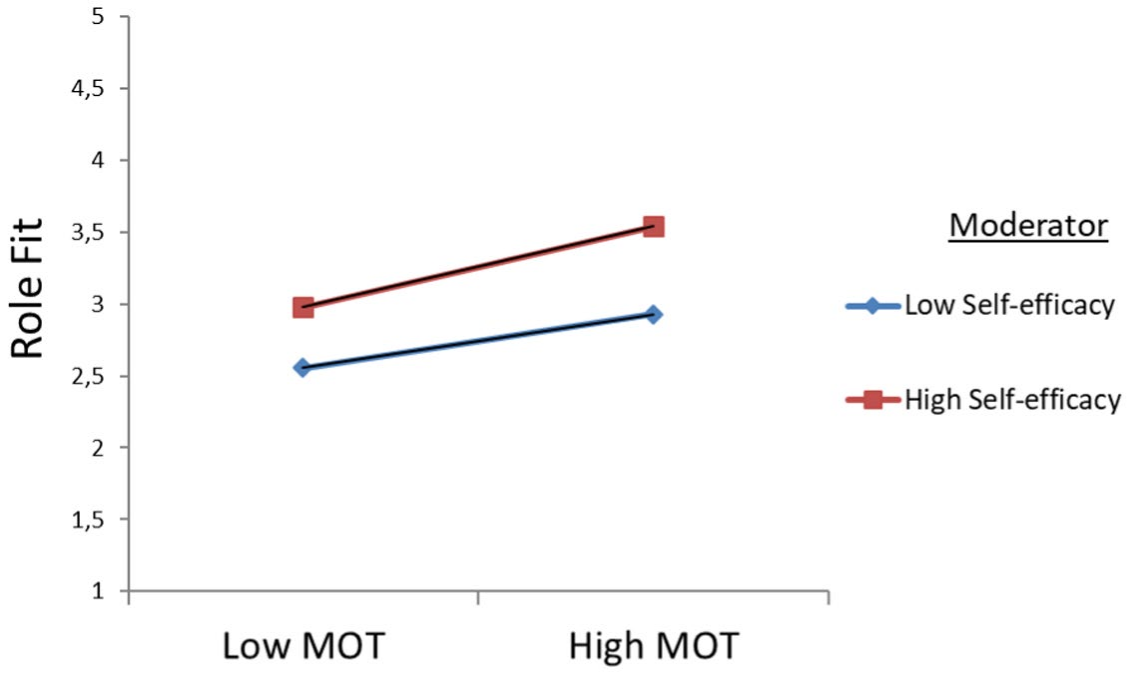
Graphical visualization of interactive effects for prosocial motivation (MOT) and self-efficacy on role fit at the beginning of military training (EMTM).

**Figure 6:**
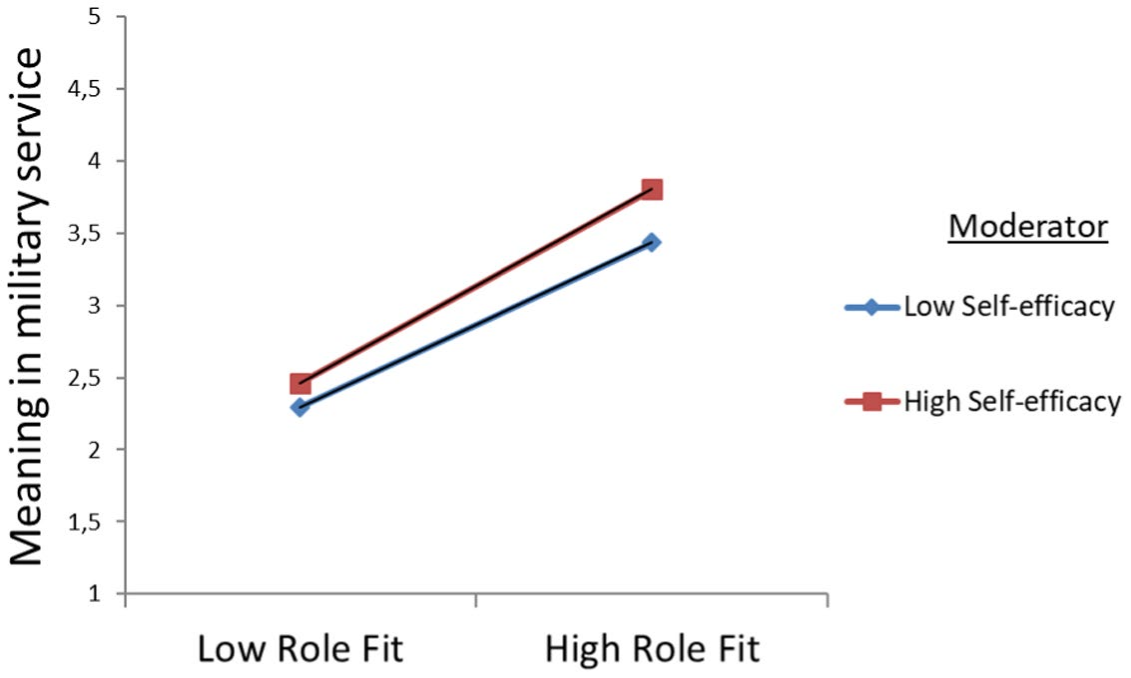
Graphical visualization of interactive effects for role fit and self-efficacy on meaning in military service at the beginning of military training (EMTM).

**Figure 7:**
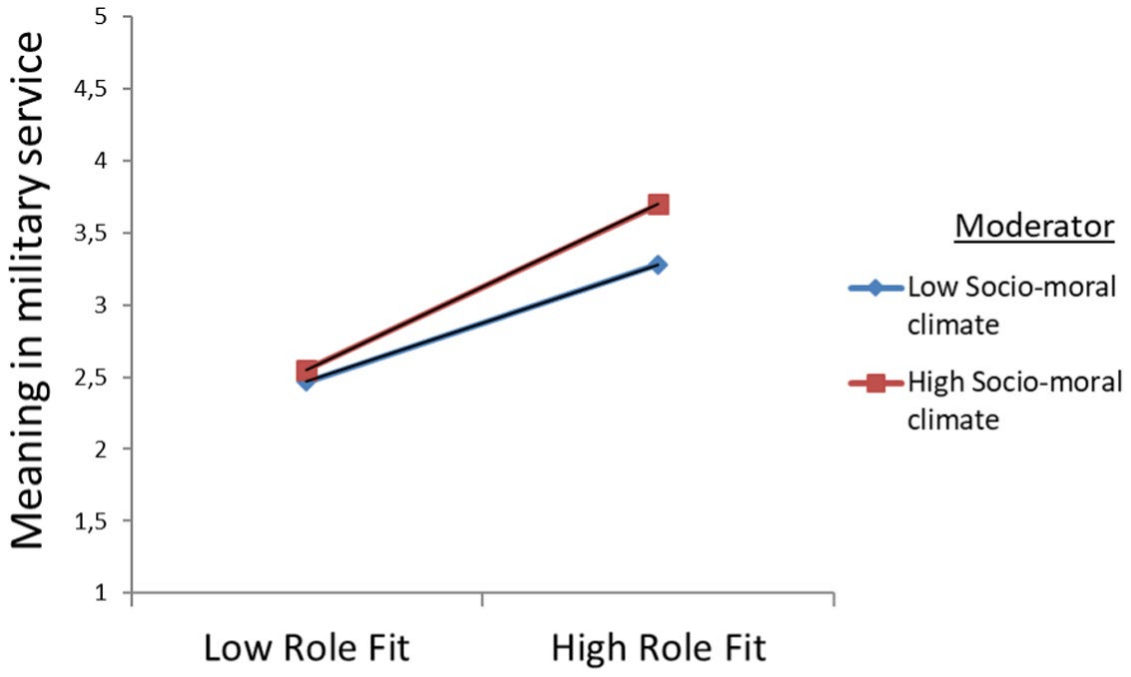
Graphical visualization of interactive effects for role fit and socio-moral climate on meaning in military service at the end of military training (EMTM).

The modeling analysis performed for the EMTM data set indicated that role fit was associated with meaning in military service (EMTM, *β* = 0.621, *p* = 0.000). Also, the moderating effect of self-efficacy on the relationship between role fit and prosocial motivation pointed to significantly positive interaction effects with: (1) prosocial motivation and self-efficacy on role fit (EMTM, MOT × SEL, *β* = 0.048, *p* = 0.019); (2) role fit and self-efficacy on meaning in military service (EMTM, RLF × SEL, *β* = 0.05, *p* = 0.039); and (3) role fit and socio-moral climate on meaning in military service (EMTM, RLF × SMC, *β* = 0.084, *p* = 0.032). The study results for EMTM are presented by simple slope graphs [low (−1 SD) vs. high (+1 SD) levels of self-efficacy] that showed the association between prosocial motivation, role fit, and socio-moral climate. The modeling allowed us to point out a significant positive association between self-efficacy and prosocial motivation, between self-efficacy and role fit, and between socio-moral climate and role fit (see Figures 5–7).

In addition, one should mention that at the beginning of military training (BMTM), the results show augmented direct positive effects of prosocial motivation on role fit and on meaning in military service for reserve soldiers with low self-efficacy. Furthermore, for reserve soldiers with higher self-efficacy, the direct effects of role fit on meaning in military service increased. Since the results of the moderation analysis disclosed positive significant interactions, the hypotheses H4a and H4b were confirmed (see Table 4, BMTM).

The results of EMTM model data set show augmented direct positive effects of prosocial motivation on role fit and on meaning in military service for reserve soldiers with low self-efficacy. Furthermore, for reserve soldiers with higher self-efficacy, the direct effects of role fit on meaning in military service increased. Subsequently, the effects of the moderation analyses revealed positive significant interactions, and let us confirm the hypotheses H4a, H4b, and H4c (see Table 6, EMTM).

**Table 6:** The moderation effects of self-efficacy construct evaluated by using the PROCESS v3.5 macro-Model 59.

	Model 4: MOT → SEL → RLF			Coeff. *β*	SE	*t*	Value of *p*	LLCI	ULCI
At the beginning of training (BMTM)	Hypothesis H4a	**Model 4 (H4a)**	Constant	0.505	0.516	0.980	0.328	−0.509	1.520
		MOT → RLF	MOT	0.305	0.169	1.801	0.037	0.046	0.638
		SEL → RLF	SEL	0.258	0.124	2.084	0.038	0.014	0.502
		MOT × SEL → RLF	MOT × SEL	0.048	0.038	1.277	0.022	0.026	0.122
		**Model 4 Summary**	***R***	***R*-sq**	**MSE**	***F***	**df1**	**df2**	**Value of *p***
			0.753	0.568	0.313	153.142	3.000	350.000	0.000
	**Model 5: MOT → SEL → RLF → MMS**			**Coeff. *β***	**SE**	***t***	**Value of *p***	**LLCI**	**ULCI**
	Hypothesis H4b	**Model 5 (H4b)**	Constant	0.462	0.483	0.957	0.339	−0.488	1.412
		MOT → MMS	MOT	0.013	0.281	0.045	0.964	−0.540	0.565
		RLF → MMS	RLF	0.813	0.311	2.617	0.009	0.202	1.423
		SEL → MMS	SEL	0.142	0.115	1.230	0.041	0.085	0.369
		MOT × SEL → MMS	MOT × SEL	0.028	0.064	1.014	0.037	0.607	0.773
		RLF × SEL → MMS	RLF × SEL	0.071	0.069	1.004	0.029	0.007	0.143
		**Model 5 Summary**	***R***	***R*-sq**	**MSE**	***F***	**df1**	**df2**	**Value of *p***
			0.849	0.720	0.247	179.216	5.000	348.000	0.000
	**Model 4: MOT → SEL → RLF**			**Coeff.**	**SE**	***t***	**Value of *p***	**LLCI**	**ULCI**
At the end of training (EMTM)	Hypothesis H4a	**Model 4 (H4a)**	Constant	0.603	0.660	0.913	0.362	−0.695	1.901
		MOT → RLF	MOT	0.234	0.220	2.063	0.048	0.199	0.667
		SEL → RLF	SEL	0.258	0.124	2.837	0.038	0.180	0.445
		MOT × SEL → RLF	MOT × SEL	0.049	0.038	1.608	0.019	0.018	0.175
		**Model 4 Summary**	***R***	***R*-sq**	**MSE**	***F***	**df1**	**df2**	**Value of *p***
			0.753	0.540	0.450	128.202	3.000	327.000	0.000
	**Model 5: MOT → SEL → RLF → MMS**			**Coeff. *β***	**SE**	***t***	**Value of *p***	**LLCI**	**ULCI**
	Hypothesis H4b	**Model 5 (H4b)**	Constant	−0.825	0.424	−1.948	0.052	−1.659	0.008
		MOT → MMS	MOT	0.620	0.180	3.452	0.001	0.267	0.973
		RLF → MMS	RLF	0.621	0.102	3.819	0.000	0.301	0.941
		SEL → MMS	SEL	0.134	0.102	1.314	0.043	0.045	0.334
		MOT × SEL → MMS	MOT × SEL	−0.101	0.041	−2.454	0.015	−0.066	−0.020
		RLF × SEL → MMS	RLF × SEL	0.050	0.037	1.378	0.039	0.002	0.122
		**Model 5 Summary**	***R***	***R*-sq**	**MSE**	***F***	**df1**	**df2**	**Value of *p***		0.909	0.827	0.183	310.203	5.000	325.000	0.000
		**Model 6: MOT → SMC → RLF → MMS**			**Coeff. *β***	**SE**	***t***	**Value of *p***	**LLCI**	**ULCI**
Hypothesis H4c		**Model 6 (H4c)**	Constant	−0.573	0.393	−1.458	0.146	−1.346	0.200
		MOT → MMS	MOT	0.529	0.176	2.999	0.003	0.182	0.876
		RLF → MMS	RLF	0.489	0.154	3.178	0.002	0.186	0.791
		SMC → MMS	SMC	0.125	0.107	1.166	0.047	0.015	0.336
		MOT × SMC → MMS	MOT × SMC	−0.096	0.045	−2.111	0.036	−0.185	−0.007
		RLF × SMC → MMS	RLF × SMC	0.084	0.039	2.154	0.032	0.007	0.161
		**Model 6 Summary**	***R***	***R*-sq**	**MSE**	***F***	**df1**	**df2**	**Value of *p***
			0.909	0.826	0.184	308.350	5.000	324.000	0.000

Model 4 = outcome variable role fit (RLF); Model 5 = outcome variable meaning in military service (MMS) and moderator self-efficacy (SEF); Model 6 = outcome variable meaning in military service (MMS) and moderator socio-moral climate (SMC). *R* = correlation coefficient and *R*-sq = correlation coefficient in square. LLCI = lower bound of 95% CI; ULCI = upper bound of 95% CI. Bootstrap sample size = 5,000.

#### 4.2.4. Moderated mediating effects

Moderated-mediation analysis was performed, and indirect pathways of mediating and moderating effects we found statistically significant. The EMTM model was evaluated for the conditional indirect effect values of the socio-moral climate by using the PROCESS v3.5 macro-Model 59 (Hayes, 2022). Given that the mediating effects of role fit differ at dissimilar values of self-efficacy and socio-moral climate; the conditional process calculates the path effects in the form of a confidence interval. The results show that the positive effect of role fit on meaning in military service was higher at higher levels of self-efficacy or socio-moral climate. Similarly, while the mediating effect of role fit on the relationship between prosocial motivation and meaning in military service was significant, the positive effect of role fit on meaning in military service decreased significantly at lower levels of self-efficacy or socio-moral climate. The conditional indirect effects presented in Table 7 confirm that if the value of self-efficacy or socio-moral climate increases, the effect of role fit will also increase, with the effect at ± 1 standard deviation for self-efficacy. The results of the analysis showed a significant positive interaction of self-efficacy in the mediation model. Accordingly, H5a, H5b and H6 can be accepted.

**Table 7:** The conditional indirect effects through role fit (RLF) evaluated by using the PROCESS v3.5 macro-Model 59.

	Mediator (RLF) MOT → RLF → MMS	Moderator (SEL) value [±SD]	^**^Effect	Boot SE	Boot LLCI	Boot ULCI
BMTM	Role fit (RLF)	3.769	0.408	0.047	0.310	0.497
	Role fit (RLF)	4.414	0.436	0.045	0.349	0.523
	Role fit (RLF)	5.000	0.462	0.055	0.363	0.575
EMTM	Role fit (RLF)	3.597	0.415	0.055	0.306	0.520
	Role fit (RLF)	4.364	0.486	0.047	0.397	0.583
	Role fit (RLF)	5.000	0.548	0.059	0.438	0.673
EMTM	Role fit (RLF)	3.125	0.479	0.064	0.357	0.608
	Role fit (RLF)	3.881	0.517	0.052	0.413	0.614
	Role fit (RLF)	4.636	0.554	0.063	0.426	0.676

Values for moderators self-efficacy (SEL) or socio-moral climate (SMC) are presented for ±one SD from the mean; ** signifies a 95% confidence interval for the mediated effect. Bootstrap sample size = 5,000.

## 5. Discussion

Finding meaning in military service helps maintain reservist motivation and commitment to military service (Gazit et al., 2021), as well as balance their civilian and military lives for a better wellbeing (Griffith, 2011). In previous studies, it was found that a military life for reservists could vary from complementary to the civil one, where service provides satisfaction that cannot be achieved in other ways, and to discordant identity where a military life conflicts with the established identity (Griffith and Ben-Ari, 2021). Research on meaning in the professional army shows that meaning is formed through identity with the military. In the case of reservists, who are not full-time soldiers and only perform their civic duty, meaning is derived from serving their country (Redmond et al., 2015). Our study refines these findings and relates it to the common concept that is used in psychology research and known as “prosocial motivation.” More specifically, our results show that prosocial motivation directly influences meaning in military service right at the beginning and the end of military training. This effect is also indirect; that is, it occurs through other subjectively perceived individual and organizational constructs.

To our knowledge, this is one of the first studies to investigate prosocial motivation as the main variable affecting meaning at work or other similar activities, and the first study to research military reservists’ prosocial motivation. We observed a considerable effect of prosocial motivation on meaning-making. Previous research has identified a range of factors crucial in explaining what determines meaning at work (Schnell and Hoffmann, 2020). This includes intrinsic personal reasons such as meaning in life (Lee, 2015) or health experience (Schnell, 2020; Lavy, 2022), as well as evaluations shaped by organizational factors such as organizational climate (Schnell, 2020; Melati et al., 2021), or work engagement (Dan et al., 2020). Our study extended this list of determinants by incorporating prosocial motivation as a variable that has an impact on meaning-making; furthermore, we have identified several indirect factors important in this process, that is, role fit within an organization, self-efficacy of the person, and organizational socio-moral support. More specifically, we indicated that at the beginning of training a part from prosocial motivation to meaning in military service displays a significant interaction between self-efficacy. The investigated indexes of moderated mediation were significant providing evidence for moderated mediation. These indirect effects are perhaps the most important implication of this study.

Meaning in military service is conceptualized in line with meaning at work, which is described as a complex process of sense-making in a specific context (Kamp, 2009). Using this framework in describing meaning in military service, the sense-making pathways and the variables influencing them become particularly important. In the military research literature, four variables are highlighted: (i) personal commitment to serve the country and its people, which, in the military literature, is codified as “warrior ethos” (Pressfield, 2011) and in the psychology literature as pro-social motivation, (ii) role fit within a military organization (Sørlie et al., 2020), (iii) soldiers’ self-efficacy, the capacity to accomplish physically and psychologically demanding tasks (Nindl et al., 2018), and (iv) a supportive social and moral environment formed by specific structure and culture in the military (Williams, 2010). In our study, we combined these four subjective factors into a pathway that leads to meaning in military service. Our study shows that role fit statistically significantly mediates the relationship between prosocial motivation and meaning in military service. We found this link both at the beginning and the end of military training. These findings are in line with an extensive scholarly literature on person-organization (P-O) fit which is found to have a significant impact on the various factors related to work attitude (see a meta-analysis of relationships between P-O and work attitudes by Verquer et al. (2003). In this context, our study extends existing research by showing a strong moderated-mediation effect of role fit. In addition, we found that the positive moderating effects of self-efficacy or social-moral climate on role fit are stronger when soldiers’ self-efficacy is greater and the organization’s social-moral support is higher. More specifically, our research indicates that prosocial motivation is positively related not only to meaning in service, but also to role fit in military organizations and the person’s self-efficacy. We found that at the end of training—when the context of the organization becomes more familiar to the individual—the importance of role fit on meaning in service has increased.

### 5.1. Limitations and future research directions

Several limitations of this study must be addressed. First, the data used in this study are composed of self-evaluation and self-perception data. As a result, subjective factors could have been underestimated or overestimated, as has other research shown (Zhang et al., 2013). To overcome this limitation, we performed Harman’s single-factor test to investigate the potential common-method variance among the study variables, and the average variance extracted (AVE) was measured to show the convergence of the construct. Second, we collected data only on one group of reservists (one intake), and therefore contextual factors in national security and geopolitical situation at the time of research may have had an impact on the general mood of reservists. The standardized training curriculum is likely to have eliminated this effect, but due to the scope limitation of this study, we were unable to verify this. We would therefore like our hypotheses to be supported by longitudinal studies in the future. Third, our research sample consisted only of men since only men are called to military reserve in Lithuania. In the previous studies, it was found that gender differences do not make a significant difference to overall meaning-making, but in individual cases, gender differences are important (Wood and Conway, 2006). Consequently, the results of the study should be interpreted in a broader context than that of army reservists; the findings could be found as gender biased.

Implications for future research. The statistical models we have built on the basis of factors identified in previous studies show high consistency. Specifically, the willingness to serve others (prosocial motivation), the role fit with the military organization, the soldiers’ self-development, and the supportive socio-moral environment in military organizations have a statistically significant effect on one another. Therefore, future research needs to focus on these factors not in isolation but in synergy while analyzing reservists and reservist military service. In addition, military service is highly related to self-construction and self-identity (Griffith and Ben-Ari, 2021); therefore, future research may focus more on what interventions are “changing” identity (Barnett et al., 2021) and contribute to meaning-making in the military context.

## Data availability statement

The raw data supporting the conclusions of this article will be made available by the authors, without undue reservation.

## Ethics statement

The study was approved by the General Jonas Zemaitis Military Academy, Protocol No. PR-1815. Informed consent was obtained from all subjects involved in the study.

## Author contributions

RK developed research instrument and executed the study. RS and SB designed the study, executed the study, and wrote the paper. SB conducted the data analyses. RK and RV collaborated with the design, execution of the study, and editing of the manuscript. ON and IM-K assisted with the editing of the final manuscript and recruitment process. All authors contributed to the article and approved the submitted version.

## Funding

This research was funded by the Research Council of Lithuania (LMTLT) under project agreement No. S-LU-22-9; the principal investigator of the grant was Svajone Bekesiene.

## Conflict of interest

The authors declare that the research was conducted in the absence of any commercial or financial relationships that could be construed as a potential conflict of interest.

## Publisher’s note

All claims expressed in this article are solely those of the authors and do not necessarily represent those of their affiliated organizations, or those of the publisher, the editors and the reviewers. Any product that may be evaluated in this article, or claim that may be made by its manufacturer, is not guaranteed or endorsed by the publisher.
